# Infant Habituation Task Performance Is Associated With Familial Risk for Attention-Deficit/Hyperactivity Disorder

**DOI:** 10.1016/j.jaacop.2026.06.003

**Published:** 2026-06-17

**Authors:** Heather M. Joseph, Anastasia N. Kerr-German, Jihui L. Diaz, Michelle A. Wilson, Erik Thiessen, Brooke S.G. Molina

**Affiliations:** a University of Pittsburgh, Pittsburgh, Pennsylvania; b Mercer University, Macon, Georgia; c University of Pittsburgh Medical Center, Pittsburgh, Pennsylvania; d Carnegie Mellon University, Pittsburgh, Pennsylvania

**Keywords:** ADHD, attention, infant, cognition

## Abstract

**Objective:**

Habituation paradigms have been widely used with neurotypical infants, distinguishing short (vs long) looking as a signal of early individual differences in attention and learning efficiency. An objective task has yet to be identified linking potential early markers of risk for childhood attention-deficit/hyperactivity disorder (ADHD) in infancy before formal identification of ADHD.

**Method:**

Infants 8 to 10 months of age from 2 cohort studies examining early signals of risk for ADHD completed 2 habituation tasks (with multimodal and unimodal stimuli). Latent growth mixture models were used to identify subclasses of looking times across 20 trials.

**Results:**

Participants were 147 infants (mean = 9 months, 52% male) at high (at least one parent with ADHD; n = 68) or low (neither parent with ADHD; n = 79) familial risk for ADHD. Three subclasses based on initial looking time and speed of habituation were identified for both tasks. Infants at familial risk for ADHD had significantly longer initial looking times to the multimodal task (z = −2.53, *p =* .01) and were 4.5 times more likely to fall into a long looker–slow to habituate subtype as opposed to the short looker subtype on the multimodal task (odds ratio = 4.5, *p* = .02).

**Conclusion:**

This study is the first to identify an objective task of infant attention and learning with performance associated with familial likelihood of childhood ADHD. Early identification of children who may develop ADHD is crucial to move toward development and implementation of preventive interventions to potentially reduce symptoms and impairment for children with ADHD.

**Diversity & Inclusion Statement:**

We worked to ensure sex and gender balance in the recruitment of human participants. We worked to ensure race, ethnic, and/or other types of diversity in the recruitment of human participants. We worked to ensure that the study questionnaires were prepared in an inclusive way. One or more of the authors of this paper self-identifies as a member of one or more historically underrepresented sexual and/or gender groups in science.

## Plain language summary

Attention-deficit/hyperactivity disorder (ADHD) is common and associated with challenges beginning as early as preschool. This study examined whether a computer task could be used to identify infants who might later develop ADHD. The authors studied 147 infants, half with parents with ADHD. Infants who had less task-related attention and took longer to complete the task were more likely to have a parent with ADHD. These findings suggest that a computer task might help identify which infants could benefit from early interventions.

Attention-deficit/hyperactivity disorder (ADHD) is the most common neurodevelopmental disorder, with an estimated worldwide prevalence of 5.9%.^1^ The economic burden associated with ADHD is estimated to be $122.8 billion per year because of impairments in social, educational, and occupational functioning.^2^ Core symptoms of ADHD include inattention, hyperactivity, and impulsivity.^3^ Inattention is thought to be one of the first identifiable symptoms to emerge with clinical significance from a very early age.^4^ Inattention can interfere with pre-academic and social skill development even before the full presentation of the disorder.^5^ Yet, no objective measures have been used to identify early signals of inattention in very young children before the clinical diagnosis is possible with current approaches.

Infants visually attend to the world around them even before they use verbal or nonverbal communication to indicate what captures their attention. Traditional infant visual attention paradigms (ie, visual paired comparison paradigms^6^, ^7^, ^8^, ^9^, ^10^) have provided rich data on how infants acquire knowledge and how the systems processing this information update across time scales (eg, moment-to-moment and across developmental periods). In this context, learning can be assessed in non-verbal children via comparing or contrasting their looking behavior when presented with familiar and unfamiliar objects. Infants have demonstrated novelty preferences followed by a transition to familiarity preferences in later infancy. These early developing attentional abilities also correspond to underlying neural systems (ie, frontal, temporal, parietal connectivity^11^^,^^12^) that later give rise to executive functioning in early life. Thus, studying visual attention in the context of novelty vs familiarity can shed light on early indicators of pathology in attentional abilities across infancy.

Habituation paradigms capitalize on novelty preference in infancy and provide a window into the attentional landscape of infants. In the context of visual attention, habituation is the temporary decrement in visual engagement with repetition of or continuous exposure to a visual stimulus.^13^ Habituation paradigms begin with a familiarization phase, during which infants attend to a novel stimulus until looking reaches a critically a low level that indicates reduced cognitive resources being allocated to processing that stimulus. The reduction in responding to a stimulus suggests that the infant has completed processing the new information and has formed a temporary memory trace or working memory for the item. Although memory clearly plays a role in habituation, the initial attentional engagement component of this cognitive process, or attentional capture, acts as the cognitive gatekeeper to individual performance differences in this task.^14^ Specifically, attention can be conceptualized as the first point in the process of habituation that, when individual differences are present, can predict later performance differences.

Individual differences in an infant’s latency to habituate within the aforementioned visual attention paradigms has been found to be predictive of later-life outcomes such as intelligence, cognitive processing efficiency in early childhood, executive functioning (eg, working memory), and standardized test scores in late childhood.^15^, ^16^, ^17^, ^18^ The latency to visually habituate serves as a behavioral marker for disengagement (ie, the ability to withdraw attention from one visual stimulus in the service of attentional shifting to a new stimulus) with visual information in the environment. Disengagement and orienting (ie, engaging with new visual stimuli) are individual processes that develop in service of broader attentional efficiency throughout infancy.^19^, ^20^, ^21^, ^22^ In addition, disengagement and subsequent orienting are critical visual attention abilities predictive of regulatory skills such as effortful control and sustained attention in the toddler years,^23^ both of which have been found to be associated with childhood ADHD.^24^ Individual differences in visual attention measured by look duration (short = low or below average, vs long = high or above average)^25^^,^^26^ in infants under 12 months of age have also been linked to broader executive functioning performance in the preschool years.^27^^,^^28^ Thus, looker status (ie, short vs long) and latency to habituate in infancy could be an indicator of potential divergence in early developing attention skills, such as those that could be seen in pathologies such as ADHD.

Decades of research have used visual attention tasks to examine cognitive development among risk groups such as premature infants, a population at risk for neurodevelopmental delays and reduced sustained attention in childhood akin to ADHD.^29^^,^^30^ Prior research has identified cognitive deficits or developmental lags in visual information processing across the first year of life for infants born prematurely. Specifically, premature infants at 4, 6, and 12 months of age have been found to have impaired visual memory and slower processing speeds compared to infants born full term.^18^^,^^31^, ^32^, ^33^ A meta-analysis of comparative studies on habituation performance by Kavšek and Bornstein further clarified that prematurity alone was not indicative of task performance.^34^ Instead, premature infants with additional medical conditions known to be associated with increased risk for neurodevelopmental disorders including ADHD,^35^ such as intraventricular hemorrhage or respiratory distress syndrome, were found to have significantly worse task performance (ie, reduced efficiency/greater number of trials to habituation)^36^ suggestive of delays in cognitive development compared to full-term infants. Albeit limited, research has found that school-age children and adolescents with ADHD habituate more slowly^37^ and have a greater preference for novel stimuli^38^ than do their typically developing peers. In addition, Miller *et al.* found that infants who would later go on to develop ADHD showed a different developmental trajectory of total looking time during 2 tasks (a social preference task and an emotion-processing task) than their typically developing peers, with longer looking times at 3 months and no increase in looking times from 3 to 24 months.^39^ In light of this literature, we suggest it is possible that visual attention and habituation task performance patterns (longer looking times and slower habituation) may be detectable in infancy and predictive of cognitive dysfunction in childhood, even before childhood ADHD can be reliability diagnosed.

Identifying children reliably for ADHD risk has thus far proved to be a challenge. To this end, an international consortium of investigators has been established to advance research on the early expression of ADHD.^40^ Given that ADHD is highly heritable, with estimates between 77% and 88%,^1^ many of the investigators in the consortium are prospectively examining cohorts of infants who are born to parents with or who have siblings with ADHD and who thus have an enhanced likelihood of childhood ADHD.^41^, ^42^, ^43^, ^44^ Recent work on early precursors for ADHD has identified “broad and non-specific phenotypic differences” among infants who later develop childhood ADHD as compared to those who do not.^40^ These include markers of neural and behavioral development (eg, motor/milestone development, temperament), none of which are objective tasks of infant attention that may be useful for screening to identify children at greatest need for early intervention.^24^^,^^45^ Theoretically, habituation has the potential to be an early signal of increased likelihood of childhood ADHD that can be objectively assessed in infancy and has yet to be tested empirically.^46^ Examining early developing attention with habituation paradigms among infants at increased familial likelihood for ADHD could serve as an initial step toward the identification of an objective marker of risk before ADHD can be clinically assessed.

### Current Approach

The current study tests the hypothesis that infant attention, specifically habituation task performance, would be less optimal in infants born to a parent with, vs without, ADHD. Using 2 habituation tasks, one with a unimodal (static visual) stimulus and one with a multimodal (dynamic visual plus auditory) stimulus, trajectories of infant looking times across trials on each task were used to examine the odds of being at high risk for ADHD based on familial ADHD (ie, having at least one parent with ADHD). This study is the first to identify subclasses of infant looking trajectories on habituation tasks that capture and characterize individual performance metrics (ie, both initial looking time and number of trials to habituate) in association with familial risk for ADHD. We hypothesized that infants at high familial risk for ADHD would demonstrate less optimal task performance (longer initial looking time and greater number of trials to habituation) suggestive of greater inattention during a habituation task.

Importantly, the multimodal condition allows for examination of attention within an integrated audiovisual context, which may place greater demands on attentional control and sensory integration processes than unimodal stimuli. Probing sensitivity to changes within such contexts may therefore reveal subtle individual differences in attentional processing that are not captured by unimodal paradigms alone. The inclusion of both unimodal and multimodal habituation conditions was theoretically motivated by the expectation that these stimulus types place different demands on infant attentional systems. Unimodal (static visual) stimuli primarily index basic visual orienting and disengagement processes, whereas multimodal (dynamic audiovisual) stimuli introduce increased perceptual complexity and require the integration of information across sensory modalities. Such multimodal contexts are thought to place greater demands on attentional control, including the ability to allocate, sustain, and shift attention in the presence of competing or redundant sensory input.

These distinctions are particularly relevant in the context of familial risk for ADHD. ADHD has been associated with alterations in attentional control, including heightened sensitivity to salient or complex stimuli, and difficulty regulating attention under increased cognitive load. As such, we anticipated that group differences would be more pronounced in the multimodal condition, in which attentional demands are higher, compared to the unimodal condition, which may be less sensitive to subtle individual differences in attentional regulation. Accordingly, we hypothesized that infants at high familial risk for ADHD would demonstrate less optimal habituation performance across both tasks, but that these differences would be amplified in the multimodal condition. Specifically, we expected longer initial looking times and slower habituation rates in high-risk infants, particularly in response to multimodal stimuli.

## Method

### Participants

Participants were infants (8-10 months of age) and their biological parent pairs recruited from southwestern Pennsylvania for participation in one of 2 cohort studies: the Pittsburgh ADHD Risk in Infancy Study (PARIS)^42^^,^^47^ or the New(born) PARIS study.^48^ These studies enrolled mother–father–infant triads from 2018 through 2024 to longitudinally examine early predictors of childhood ADHD onset. Families with infants (infant age at enrollment: 6-10 months for cohort 1 and less than 4 weeks of age for cohort 2) in which both biological parents were willing to participate were eligible for enrollment. Infants were classified as either high familial risk (at least one parent with ADHD) or low familial risk (neither parent with ADHD) for ADHD based on parental ADHD (*DSM-5* criteria for childhood ADHD with persistence into adulthood). Both male and female infants were enrolled, and sex effects were considered in recruitment balancing sex across risk groups. To reduce overlap between preterm infant status and risk for ADHD, families were excluded if the infant was born before 37 weeks of gestation, had a low birth weight (<5 lb 8 oz), or was prenatally exposed to substances (tobacco, alcohol, marijuana, benzodiazepines, opioids, or medication-assisted treatment for opioids) as determined by both mother and father report.

### Procedures

All parents first participated in a research visit to determine ADHD status and co-occurring psychiatric disorders. Assessments were conducted by a masters- or doctoral-level clinician, research trained to evaluate symptoms and impairment of ADHD across the lifespan using the Conners’ Adult ADHD Diagnostic Interview (CAADID).^49^ Parents also completed questionnaires remotely using Qualtrics (Provo, UT) regarding demographics. Infants completed 2 conditions (unimodal and multimodal stimuli) of a habituation task while seated in a parent’s lap or an infant seat (with the parent out of sight) if the parents requested. Parents were instructed to help keep the infant in their lap, but to refrain from directing their child’s attention or interacting with their child during the task. Dark glasses and headphones with music were provided to parents to avoid parents unintentionally providing feedback to the infant regarding the task. The infants’ familial risk status and study hypotheses were masked from the examiner to reduce risk of bias. The examiner was out of the infant’s sight for the duration of the task. PARIS participants completed the habituation task in the laboratory (n = 72), whereas New(born) PARIS participants (n = 75) completed the task in their home. Infants with complete data on either task condition were included in the analyses. Informed consent was obtained before study participation. All families were compensated for their time and the study protocol was approved by the University of Pittsburgh Institutional Review Board.

### Assessments

#### Habituation Task

The habituation (familiarization) phase comprised up to 20 trials. The trial design was infant led and individually normed such that the familiarization phase of the tasks ended after the infant habituated to the stimulus (look time decreased by 50% from their average look time during the first 3 trials) or after 20 trials occurred (accumulated 400 seconds of stimulus presentation), whichever occurred first. Before each trial, an “attention-getter” animated clip of Winnie the Pooh appeared centrally on the screen, along with audio of a female voice on loop saying, “Look, it’s Winnie the Pooh! Good baby!” After the attention-getter attracted the attention of the child (ie, the child fixated their gaze upon the screen), the experimenter manually began a new trial. The familiarization phase stimulus was an image of thin, rainbow-colored vertical lines on a white background for the unimodal condition, and a looming green circle on a black background paired with a “KOO” sound (synthesized speech) for the multimodal condition (Figure 1). Once the infant looked away for more than 2 seconds or the maximum trial duration (20 seconds) was reached, the attention-getter clip would play, and the experimenter triggered the next trial once the child was again centrally fixated.

**Figure 1:**
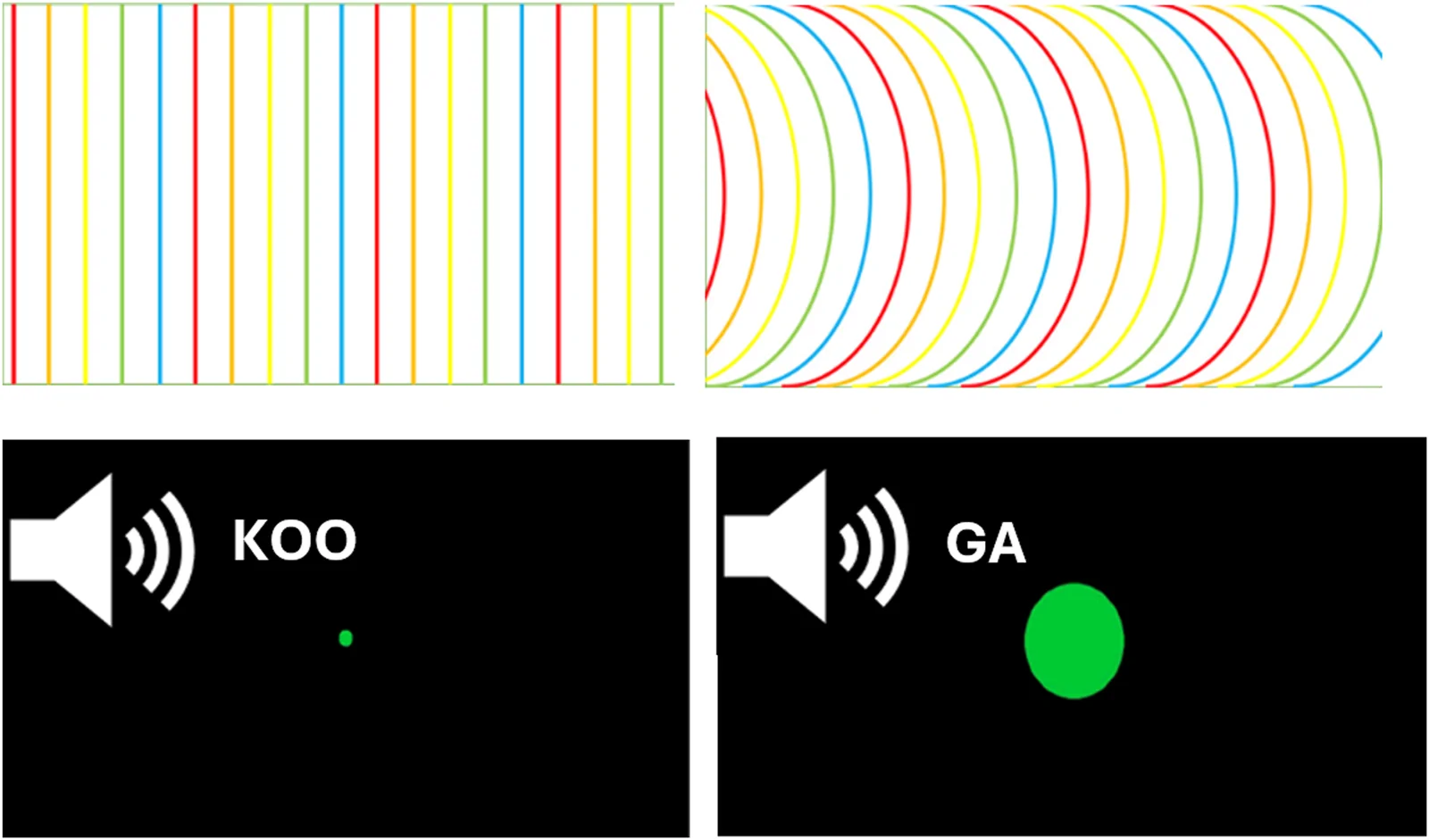
Habituation and Dishabituation Stimuli ***Note:****Unimodal (static visual) task pictured above; multimodal (auditory + dynamic (moving) visual) task pictured below.*

The dishabituation phase followed immediately after the familiarization phase and comprised 3 trials with a novel stimulus. Dishabituation occurs when looking at the novel stimuli compared to the previously habituated stimuli (novelty preference) is longer. Dishabituation demonstrates not only the efficiency of the initial attention processing (ie, rate to habituate during the familiarization period) but also perseverance of that memory trace in the testing phase.^50^ Like the familiarization phase, each trial was shown until the child looked away for 2 seconds or the maximum trial time of 20 seconds was reached. During the dishabituation phase, infants were shown 3 novel trials, consisting of a new unimodal (curved lines instead of straight) or multimodal (the same looming green circle paired with a “GA” sound) stimulus to test dishabituation. It should be noted that the primary difference between the unimodal and multimodal stimulus is that the novelty of the stimuli was specific to the modality of the sensory information provided. The unimodal stimulus was novel in visual features, whereas the multimodal stimulus was novel in its auditory, but not visual, features (ie, same looming green circle as in the familiarization phase). As this is an infant habituation paradigm measuring visual attention, an auditory-only stimuli comparison would not be feasible. The unimodal and multimodal stimuli were not explicitly matched on perceptual complexity. The multimodal stimulus inherently included dynamic visual motion and auditory input, whereas the unimodal stimulus consisted of static visual features, reflecting a contrast between lower- and higher-complexity sensory environments.

Both habituation tasks were coded in real time by a trained research specialist; the experimenter coded in real time whether the child was looking at the stimulus presented on the screen. All experimenters were trained to reliability before they could administer the task. Reliability was assessed via a set of training videos, recordings of participants in prior habituation studies. To be certified as reliable, examiners had to code a randomly selected test video such that their judgment for all look durations was within 200 milliseconds of the timing indicated by a prior consensus coding of the video.

### Data Analysis

Latent growth mixture models (LGMMs)^51^^,^^52^ were used to detect subgroups with distinctive patterns of change in looking time over the approximately 20 trials from the habituation phase for infants who habituated to the stimuli. LGMMs are multilevel models that examine variability in repeated measures within individuals to detect within-subgroup latent changes over time using linear mixed models. These models identify statistically distinct subgroups with unique trajectories of measurements with subgroup-specific linear mixed models. We fitted LGMMs with 2, 3, 4, and 5 subgroups and compared them using the Bayesian information criterion (BIC). BIC is an information criterion with a penalty for model complexity, considering both the goodness of fit and the parsimony of the model.^53^ Unimodal and multimodal tasks were analyzed separately, and 3 subgroups were identified for each task. No multiple correction was applied for analyses of 2 different tasks. The median and interquartile range (IQR) were reported for continuous variables, and the frequency and percentage were reported for categorical variables, respectively, as subgroup-specific summary statistics. The Fisher exact test and Kruskal–Wallis tests were used to examine the association of estimated habituation subgroup membership with demographic and clinical variables. Logistic regression models were fitted to investigate statistical prediction of ADHD familial risk status from estimated habituation subgroup membership. Both unadjusted and adjusted odds ratio (ORs) and corresponding 95% confidence intervals were reported. The adjusted effect of habituation subgroup membership was estimated alongside inclusion of infant sex and parent education level.

Infant sex was controlled for in all analyses. Rates of ADHD diagnoses have been reported to differ by sex, with a male-to-female ratio of 3:1^54^; prior research has confirmed differences in symptom severity, which may be due to differential genetic and cognitive labilities.^55^ In addition, research has found differences in brain activity suggesting different neural profiles by sex emerge across critical attention and executive functioning regions in children with ADHD.^56^, ^57^, ^58^ Parent education was also a covariate in the analyses as a marker of socioeconomic status and because of the association with early child cognitive development as well as later ADHD symptoms.^59^^,^^60^ All of the analyses were conducted using R statistical software version 3.6.1 (Vienna, Austria),^61^ and a 2-sided *p* value of less than .05 was considered statistically significant.

## Results

Of the 147 infant participants, 68 (46%) were at high familial risk for ADHD. Participant characteristics are shown in Table 1. Only 13 infants did not provide data for one of the 2 tasks (unimodal, n = 7; multimodal, n = 6) for the following reasons: the task was ended early because of infant fussiness (ie, crying, n = 5); an error occurred with task administration (ie, technical or experimenter error, n = 6); or the infant was distracted and did not participate in the task (ie, turning around to look at parent/glasses, n = 2). Task completion was not related to infant familial risk for ADHD (Fisher exact test *p* = .38). As expected, nearly all infants habituated in the familiarization phase (unimodal: 87.1%, n = 128; multimodal: 94.6%, n = 139). Of those infants who habituated, most dishabituated as evidenced by longer looking times when presented with novel stimulus during the dishabituation phase as compared to the habituated stimuli (unimodal: 78.1%, n = 100, *z =* −6.93, *p* < .001; multimodal: 56.8%, n = 79, *z* = −2.97, *p* = .003). Initial looking time (average of first 3 trials) to the familiarization stimuli was significantly different between stimuli modalities (unimodal: mean = 6.90 seconds, multimodal: mean = 15.96 seconds; Wilcoxon signed rank test statistic *z =* −9.77, *p* < .001).

**Table 1:** Participant Characteristics

	High-Risk (n = 68)	Low-Risk (n = 79)	*p*
	Median (IQR)	Median (IQR)	
Child age, mo	9.2 (8.6-10.3)	9.3 (8.9-10.2)	.508
Child sex	n (%)	n (%)	.869
Female	32 (47.1)	39 (49.4)	
Male	36 (52.9)	40 (50.6)	
Child race			.492
Asian	1 (1.5)	5 (6.3)	
Black or African American	5 (7.4)	7 (8.9)	
More than one race	7 (10.3)	7 (8.9)	
White or European American	55 (80.9)	60 (75.9)	
Child ethnicity			.842
Hispanic or Latino	6 (8.8)	5 (6.3)	
Not Hispanic or Latino	61 (91.2)	73 (93.7)	
Parent education			.594
Less than a 4-y college degree	12 (17.6)	13 (16.5)	
4-y College degree	20 (29.4)	18 (22.8)	
Graduate degree	36 (52.9)	48 (60.8)	

Note: IQR = interquartile range.

High-risk infants had significantly greater initial looking times on the multimodal task compared to the low-risk infants (high risk: mean = 16.84 milliseconds; low risk: mean = 15.21 milliseconds; Wilcoxon rank sum test statistic *z =* −2.53, *p* = .01). However, high- and low-risk infants did not differ on initial looking times on the unimodal task (high risk: mean = 7.01 milliseconds; low risk: mean = 6.78 msec; *z* = −0.34, *p* = .73), nor did they differ on any other task performance variables (yes/no habituated and dishabituated, number of trials to habituation, average looking time of last 3 habituation trials, average looking time of dishabituation trials).

Three classes of looking-time trajectories across the habituation task (familiarization phase) were identified in the subgroup analyses for each task condition (unimodal [Figure 2] BIC = 7621.8, entropy = 0.82; multimodal [Figure 3] BIC = 7636.4, entropy = 0.69). The classes were named short vs long lookers based on average duration of looking across the first 3 trials in relation to the sample mean. The unimodal condition classes included the following: class 1: short lookers (n = 102); class 2: long lookers (n = 19); and class 3: long lookers fast to habituate (n = 19). The multimodal condition classes included the following: class 1: short lookers (n = 23); class 2: long lookers fast to habituate (n = 92); and class 3: long lookers slow to habituate (n = 26). The classes within each condition did not differ in regard to parent education. Classes in both conditions differed in the number of trials to habituation, average looking time across the first 3 trials, and average looking time across the dishabituation trials (Table 2, Table 3). The classes in the unimodal condition also differed significantly on infant sex, whereas the classes in the multimodal condition differed on location (laboratory vs home) (Table 3).

**Figure 2:**
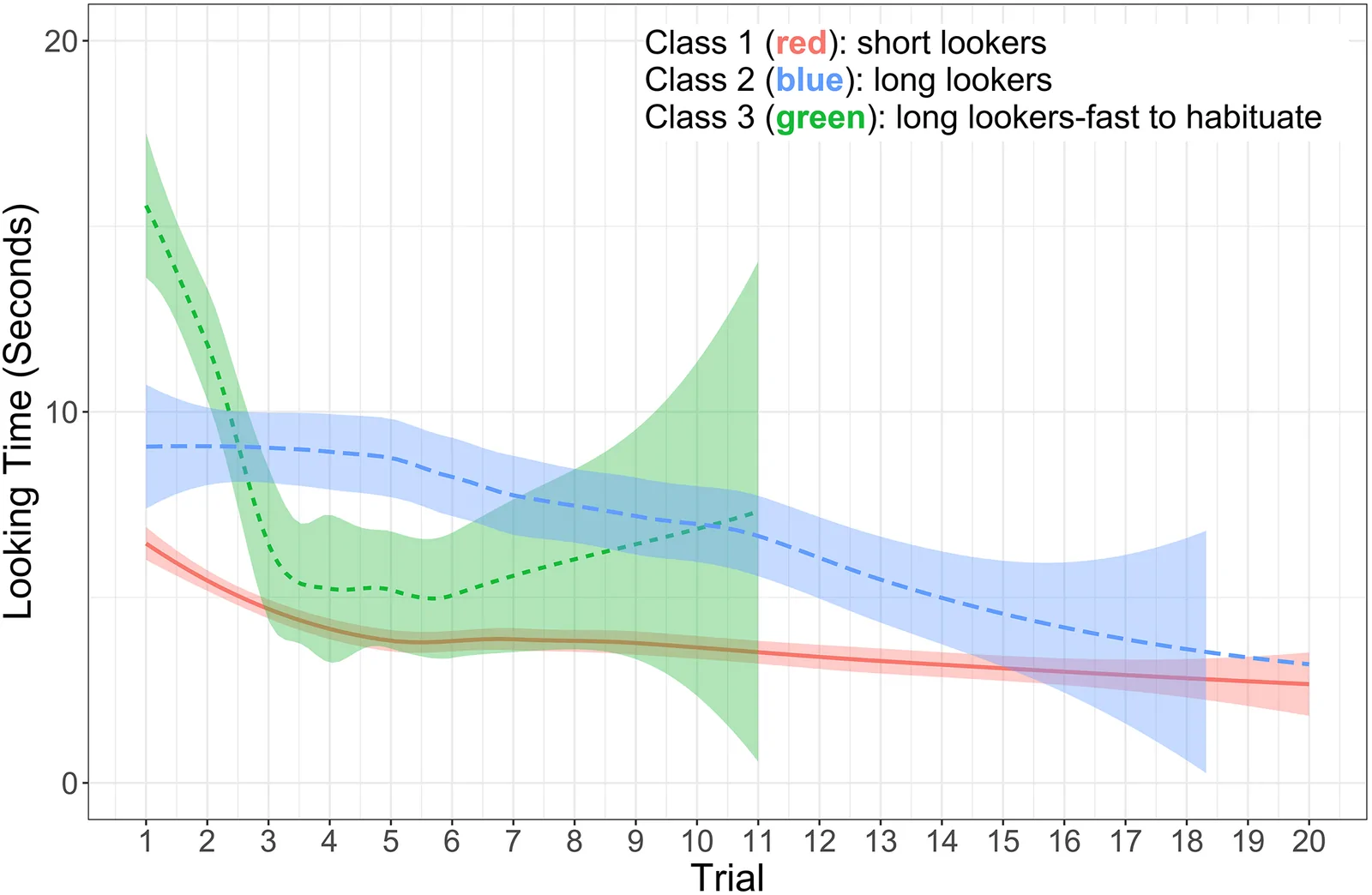
Unimodal (Static Visual) Task ***Note:****The average duration of each trial among individuals in the three classes are plotted. Lines represent the class-specific average calculated using locally estimated scatterplot smoothing (LOESS) and the shaded area represents the 95% confidence interval for the fitted LOESS curve.Class 1 (red): short lookers. Class 2 (blue): long lookers. Class 3 (green): long lookers-fast to habitutate.*

**Figure 3:**
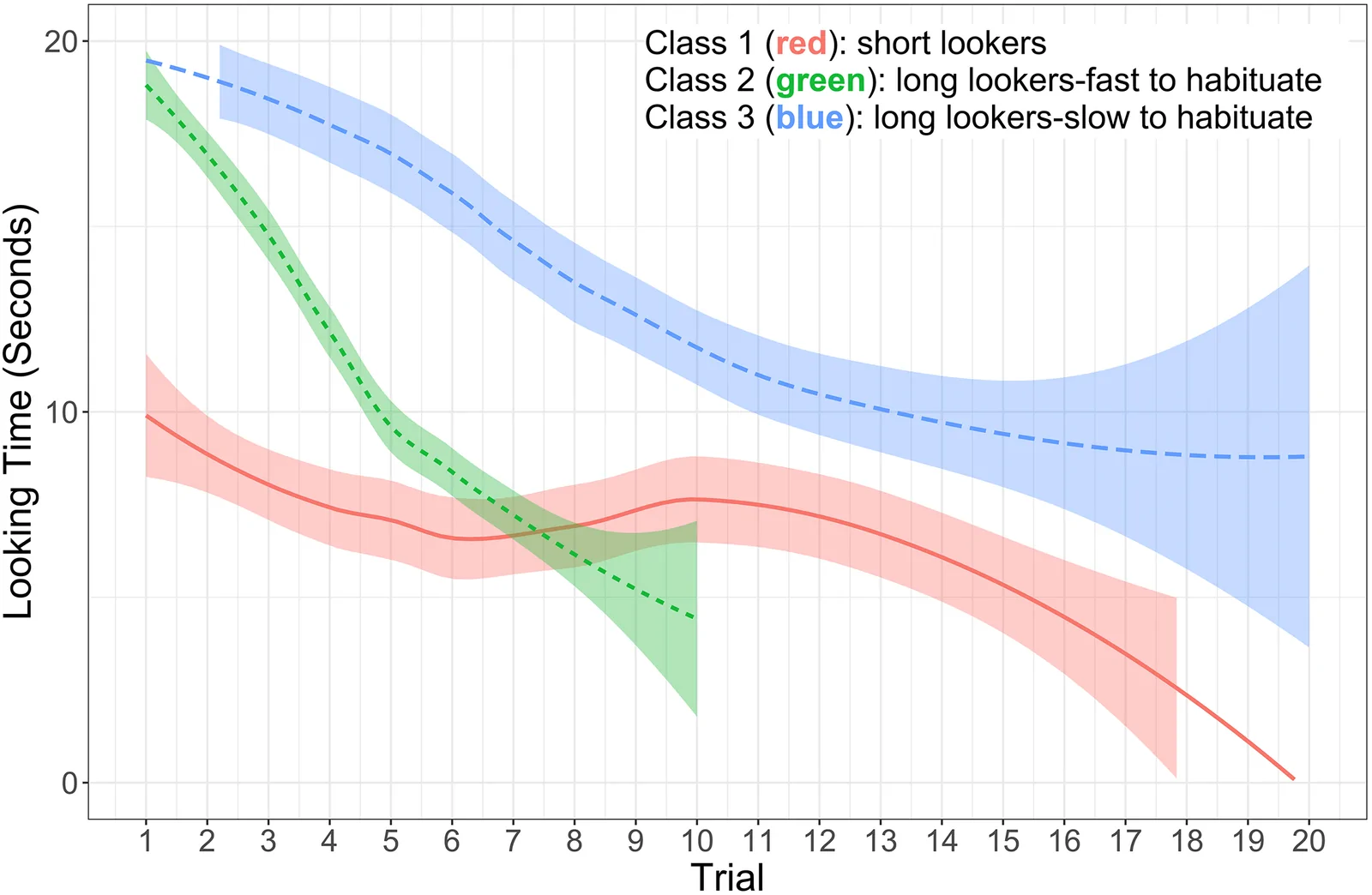
Multimodal (Auditory + Dynamic Visual) Task ***Note:****The average duration of each trial among individuals in the three classes are plotted. Lines represent the class-specific average calculated using locally estimated scatterplot smoothing (LOESS) and the shaded area represents the 95% confidence interval for the fitted LOESS curve.Class 1 (red): short lookers. Class 2 (green): long lookers-fast to habituate. Class 3 (blue): long lookers-slow to habitutate.*

**Table 2:** Unimodal Task Descriptive Statistics and Class Comparisons

	Total		Class 1 short lookers		Class 2 long lookers		Class 3 long lookers fast to habituate		*p*
	N	Freq (%) Median (IQR)	n	Freq (%) Median (IQR)	n	Freq (%) Median (IQR)	n	Freq (%) Median (IQR)	
ADHD risk status	140	67 (47.9)	102	47 (46.1)	19	8 (42.1)	19	12 (63.2)	.367
Infant sex	140		102		19		19		<.001∗
Male		73 (52.1)		45 (44.1)		18 (94.7)		10 (52.6)	
Female		67 (47.9)		57 (55.9)		1 (5.3)		9 (47.4)	
Parent education	140		102		19		19		.624
Less than a 4-y degree		24 (17.1)		17 (16.7)		2 (10.5)		5 (26.3)	
4-y College degree		36 (25.7)		25 (24.5)		7 (36.8)		4 (21.1)	
Graduate degree		80 (57.1)		60 (58.8)		10 (52.6)		10 (52.6)	
Location	140		102		19		19		.631
Home		71 (50.7)		52 (51)		11 (57.9)		8 (42.1)	
Laboratory		69 (49.3)		50 (49)		8 (42.1)		11 (57.9)	
Condition order	140		102		19		19		.074
Unimodal first		75 (53.6)		54 (52.9)		14 (73.7)		7 (36.8)	
habituated	140	128 (91.4)	102	91 (89.2)	19	18 (94.7)	19	19 (100)	.392
Trials to habituation	128	8 (6-12)	91	8 (6-12.5)	18	11.5 (11-13.8)	19	5 (5-6.5)	<.001∗
Average first 3 trials	140	6.4 (4.6-8.4)	102	5.5 (4.3-6.7)	19	9 (7.8-10.5)	19	11.2 (9.7-13.6)	<.001∗
Average dishabituation trials	128	3.8 (2.8-5.2)	91	3.3 (2.6-4.8)	18	4.5 (4-6.1)	19	5 (3.7-7.7)	.004∗

Note: Median and interquartile range are calculated for a numeric predictor, and frequency and relative frequency (%) are reported for a categorical variable. Kruskal–Wallis tests and Fisher exact tests are used to test the association of engagement phenotypes with numeric and categorical predictors, respectively. ADHD = attention deficit/hyperactivity disorder; Freq = frequency; IQR = interquartile range.

∗ *p* < .05.

**Table 3:** Multimodal Task Descriptive Statistics and Class Comparisons

	Total		Class 1 short lookers		Class 2 long lookers fast to habituate		Class 3 long lookers slow to habituate		*p*
	n	Freq (%) Median (IQR)	n	Freq (%) Median (IQR)	n	Freq (%) Median (IQR)	n	Freq (%) Median (IQR)	
ADHD risk status	141	65 (46.1)	23	6 (26.1)	92	43 (46.7)	26	16 (61.5)	.044∗
Infant sex	141		23		92		26		.071
Male		74 (52.5)		7 (30.4)		53 (57.6)		14 (53.8)	
Female		67 (47.5)		16 (69.6)		39 (42.4)		12 (46.2)	
Parent education	141		23		92		26		.593
Less than a 4-y degree		22 (15.6)		4 (17.4)		12 (13.0)		6 (23.1)	
4-y College degree		37 (26.2)		4 (17.4)		26 (28.3)		7 (26.9)	
Graduate degree		82 (58.2)		15 (65.2)		54 (58.7)		13 (50)	
Location	141		23		92		26		.003∗
Home		73 (51.8)		15 (65.2)		52 (56.5)		6 (23.1)	
Laboratory		68 (48.2)		8 (34.8)		40 (43.5)		20 (76.9)	
Condition order	141		23		92		26		.547
Multimodal first		67 (47.5)		13 (56.5)		41 (44.6)		13 (50)	
habituated	141	139 (98.6)	23	22 (95.7)	92	92 (100)	26	25 (96.2)	.119
Trials to habituation	139	8 (6-10)	22	10 (7-14.5)	92	7 (6-8)	25	12 (10-14)	<.001∗
Average first 3 trials	141	17.2 (13.4-19.9)	23	8.2 (6.9-10.1)	92	17.6 (14.8-19.9)	26	19.9 (18.5-19.9)	<.001∗
Average dishabituation trials	139	6.8 (4.8-10)	22	4.5 (3.2-6.9)	92	7.1 (5.2-10)	25	9.1 (6.3-11.9)	**<**.001∗

Note: Median and interquartile range are calculated for a numeric predictor and frequency, and relative frequency (%) are reported for a categorical variable. Kruskal–Wallis tests and Fisher exact tests are used to test the association of engagement phenotypes with numeric and categorical predictors, respectively. ADHD = attention-deficit/hyperactivity disorder; Freq = frequency; IQR = interquartile range.

∗ *p* < .05.

Infant sex, parent education, and most individual measures of task performance were not significantly associated with familial risk for ADHD (Table 4). Longer looking times for the average first 3 trials of the habituation phase of the multimodal condition were significantly associated with greater odds of being high familial risk for ADHD (odds ratio [OR] = 1.10, 95% CI = 1.02-1.20, *p* = .02). In the multimodal condition, long looker, slow-to-habituate infants (class 3) were at 4.53 times increased odds (95% CI = 1.39-16.37, *p* = .02) of being high familial risk than short looker (class 1) infants. These results remained significant (OR = 4.50, *P* = .02) after adjusting for infant sex and parent education (Table S1, available online).

**Table 4:** Univariate Logistic Regression: Prediction of ADHD Familial Risk Status

	OR (95% CI)	*p*
Infant sex (female vs male)	0.91 (0.48, 1.74)	.786
Parent education		
4-y Degree vs less than a 4-y degree	1.20 (0.44, 3.34)	.719
Graduate degree vs less than a 4-y degree	0.86 (0.35, 2.11)	.736
Location (laboratory vs home)	1.34 (0.70, 2.56)	.375
Condition order (unimodal vs multimodal first)	1.62 (0.85, 3.14)	.148
Unimodal		
Habituated	0.91 (0.27, 3.05)	.877
No. of trials until habituation	0.99 (0.91, 1.08)	.897
Average first 3 trials	1.02 (0.92, 1.15)	.672
Average dishabituation trials	1.00 (0.86, 1.17)	.985
Unimodal classes		
Class 2 vs 1	0.85 (0.31, 2.28)	.750
Class 3 vs 1	2.01 (0.74, 5.78)	.177
Multimodal		
Habituated	0.85 (0.03, 21.87)	.911
No. of trials until habituation	0.96 (0.86, 1.07)	.500
Average first 3 trials	1.10 (1.02, 1.20)	.024∗
Average dishabituation trials	1.06 (0.96, 1.17)	.236
Multimodal classes		
Class 2 vs 1	2.49 (0.94, 7.41)	.079
Class 3 vs 1	4.53 (1.39, 16.37)	.015∗

Note: ADHD = attention-deficit/hyperactivity disorder; OR = odds ratio.

∗ *p* < .05.

## Discussion

This is the first study to examine habituation performance in infancy as an early objective marker of increased risk for childhood ADHD. Three subclasses of looking trajectories were identified for both the unimodal and multimodal habituation tasks. Familial risk for ADHD was associated only with performance on the multimodal habituation task. Infants at high familial risk for ADHD had significantly longer initial looking times on the multimodal task. Infants with a combination of long looking and slow habituation on the multimodal task were at significantly greater odds of being at high familial risk for ADHD as compared to short lookers. These findings have partially supported our hypotheses that infants with task performance representing the greatest inattention (long looking and slow habituation) were at higher odds of being offspring of parents with ADHD. Notably, the finding that familial risk for ADHD was associated with performance in the multimodal, but not the unimodal, condition is consistent with our theoretical expectation that increased attentional demands would amplify individual differences in early attentional regulation. The multimodal condition, which requires processing of dynamic audiovisual input, may place greater demands on attentional control systems than the unimodal condition, thereby revealing differences that are not detectable under lower-demand conditions. From a developmental perspective, attentional processing in infancy is shaped by both bottom-up salience and emerging top-down control, with more complex or multimodal stimuli placing greater demands on these developing systems.

The novel assessment of trajectories that include both looking status and number of trials to habituation, as opposed to a single metric of habituation task performance (eg, long vs short looking status or yes/no habituation), may be better at distinguishing individual differences. Prior work with premature infants, a group at elevated risk for neurodevelopmental disorders, including ADHD, has shown inconsistent findings on habituation task metrics when conducting group comparisons by aggregate task performance values (eg, average looking time).^34^ This is likely due to the wide range of neurodevelopmental outcomes (eg, neurotypical development to severe cognitive delays) among infants born prematurely. By simply averaging task performance for the group, the ability to detect which children will go on to have the outcome is lost. Just as not all infants born prematurely will go on to develop ADHD, not all children born to parents with ADHD will develop the disorder. The identification of 3 classes of task performance was unexpected, based on previous work that focused primarily on long vs short lookers. It is possible that the intermediate class (long lookers fast to habituate) in the multimodal condition that was found to be at marginally significantly greater odds of familial ADHD might be composed of infants at familial risk with resilience factors that may protect from later development of childhood ADHD. In addition, later exploration of the emergent attentional differences in toddlerhood, as initially indexed by this task, with paradigms such as the visual attention processing protocol task^62^ could shed additional light on this third group, as divergence in attentional abilities unfolds over time in the context of higher-order executive functioning. A robust literature has identified neurocognitive performance in middle childhood and adolescence as an endophenotype for ADHD with genetic vulnerability, such that siblings of children with ADHD who do not have the disorder themselves may perform at an intermediate level between their siblings with ADHD and unaffected probands.^63^, ^64^, ^65^

An important consideration in interpreting the multimodal condition is that dishabituation was driven by a change in the auditory modality, whereas visual input remained constant. As such, this paradigm does not assess fully symmetric multimodal novelty but, rather, sensitivity to auditory change within an established audiovisual context. This design was chosen to isolate modality-specific processing while preserving the integrated nature of the stimulus; however, it also raises the possibility that auditory and visual novelty may differentially engage attentional systems. Future work systematically varying the modality of change (eg, visual-only or fully cross-modal dishabituation) will be important for disentangling modality-specific contributions to infant attention in this paradigm.

Although 3 classes were identified in each of the 2 task conditions, performance across the 2 conditions varied in meaningful ways, likely because of the stimuli presented. The dynamic visual plus auditory stimuli of the multimodal condition of the habituation task captured the attention of the infants for longer times at the beginning of the task. As such, the initial looking times for the unimodal condition classes were 5 to 11 milliseconds, whereas the short-looker class in the multimodal condition had a mean of 8 milliseconds of initial looking time. These low initial looking times in the unimodal task may have made it more challenging for the infants, particularly in the short looker class, to habituate quickly (ie, drop the looking time to less than 50% of the initial looking time, calculated as the average looking time across the first 3 trials). At 9 months of age, executive attention and underlying frontal lobe activity are emergent.^66^, ^67^, ^68^ The unimodal stimuli may not be developmentally appropriate or perceptually complex enough to sufficiently engage and sustain attention at this age. For the multimodal task, cluster membership was associated with testing location, suggesting that the multimodal task performed differently in the 2 settings. Specifically, infants in class 3 (long looker slow to habituate) were less likely to have been administered the task in the home. It is possible that infants are less interested in the stimuli in the home where there are more potential distractors than in the laboratory setting. The appropriateness of the task stimuli by developmental age and setting are critically important considerations for future research on early markers of risk for childhood ADHD.

Differences in performance across the unimodal and multimodal conditions likely reflect, in part, differences in stimulus complexity. The multimodal stimulus incorporated both dynamic visual motion and auditory input, whereas the unimodal stimulus was static and visually simpler. As expected, the higher-complexity multimodal stimulus elicited longer initial looking times, consistent with literature demonstrating that increased perceptual richness enhances attentional capture in infancy. Importantly, this difference in complexity is not orthogonal to the construct of interest, as real-world attentional demands are inherently multimodal and dynamically complex. At the same time, we acknowledge that differences in perceptual complexity may have contributed to observed differences in habituation trajectories independent of familial ADHD risk. As such, findings from the multimodal condition should be interpreted within the context of a higher-complexity attentional environment rather than as directly comparable to the unimodal condition. Future work systematically equating or parametrically manipulating stimulus complexity across modalities will be important for disentangling the relative contributions of sensory complexity and attentional processing differences.

This research study has notable strengths, including the infant sample enhanced for likelihood of ADHD, with both biological parents rigorously evaluated for childhood and adulthood ADHD diagnoses, as well as the use of a validated task of habituation with comparison of unimodal and multimodal stimuli. However, the findings and future potential need to be considered in the context of the study’s limitations and need for replication. Albeit promising, it is important to realize that these classes are associated with increased odds of familial likelihood and not childhood ADHD. Longitudinal follow-up of these infant cohorts is ongoing to determine which children develop the disorder.

Several limitations of the present study should be considered, particularly with respect to the interpretation and generalizability of the findings. First, risk status was defined based on familial liability rather than confirmed clinical outcomes in the child. Although familial risk is a well-established and informative proxy in developmental research, it does not equate to eventual diagnosis, and considerable heterogeneity exists in developmental trajectories among high-risk infants. As such, the present findings should be interpreted as reflecting early markers associated with elevated risk, rather than as definitive predictors of later ADHD. Longitudinal follow-up linking these early measures to clinical outcomes will be critical for establishing predictive validity. In addition, parents with ADHD, and their partners, have higher rates of depression and anxiety.^69^ Thus, future research should examine the specificity of these findings for infants at familial risk for ADHD as opposed to risk for another psychopathology or reared by parents with depression and/or anxiety. Next, subgroup sample sizes were modest, particularly when stratifying by risk status and experimental condition. This may have limited statistical power to detect smaller effects, and increases the possibility that observed effects are sample specific. In addition, smaller subgroup sizes may reduce the stability of parameter estimates and constrain the generalizability of the findings to broader populations. Replication in larger, more diverse samples will be important for confirming the robustness of these effects. Taken together, these considerations highlight the importance of interpreting the present findings as preliminary but informative evidence of early attentional differences associated with familial risk for ADHD, pending further validation in longitudinal and larger-scale studies.

The mechanism underlying habituation is complex, and is theorized to include “multiple mechanisms” across neuronal processes and networks to enact behavioral changes.^70^^,^^71^ An important consideration in interpreting these findings is the developmental timing of the neural systems implicated in ADHD. ADHD has been consistently linked to atypical functioning in frontal and frontostriatal networks, particularly those supporting attentional control and executive processes.^72^^,^^73^ However, during the 8- to 10-month age range examined in the present study, these frontal systems are only beginning to emerge and are not yet fully functional. As such, habituation paradigms administered at this age may be operating at the upper boundary of developmental appropriateness for capturing individual differences related to later ADHD risk.

Within this context, the differential predictive utility of the multimodal—but not the unimodal—habituation condition may reflect differences in task sensitivity rather than a general superiority of multimodal stimulation. Specifically, unimodal habituation at this age may not place sufficient demands on emerging attentional control systems to reveal meaningful variability associated with risk status. In contrast, multimodal stimuli likely impose greater integrative and attentional demands, thereby more effectively engaging nascent frontal networks. Thus, the present findings may be better interpreted as evidence that multimodal habituation paradigms constitute a more developmentally appropriate measure of early attentional processing at this age, rather than as evidence of inherent superiority of multimodal over unimodal stimulation for prediction. Future work should systematically vary task complexity across developmental stages to determine when unimodal vs multimodal paradigms most effectively capture emerging risk-related differences in attentional processing.

Infants at elevated likelihood of neurodevelopmental disorders or for whom clinicians have identified clinical concerns, even before they display developmental delays, may be eligible for early intervention services through the Individuals with Disabilities Act (IDEA) Part C.^74^ Universal screening for autism spectrum disorder (ASD) and developmental delays across the first 3 years of life in primary care is recommended by the American Academy of Pediatrics^75^^,^^76^ to facilitate referral to early intervention services that have been found to change parenting behaviors and to improve child outcomes in a range of domains including cognitive, language, motor, and socioemotional skills as well as severity of ASD symptoms.^77^^,^^78^ Although behavioral and cognitive markers of later development of ADHD have been found to be present early in life, very few have been examined in infancy or toddlerhood,^24^^,^^43^^,^^45^ and current behavioral screeners are not sensitive enough to differentiate children at increased likelihood of developing ADHD.^79^ Thus, identification of an objective, non-verbal, computer-based task to measure early attentional difficulties may one day facilitate earlier identification and preemptive intervention for young children at elevated likelihood of developing ADHD, an approach also currently being used with infants and toddlers at risk for autism spectrum disorders examining eye-gaze to social stimuli.^80^ Moreover, the adaptability of the habituation task for administration outside of the laboratory in the current study underscores its potential utility in improving access to developmental risk screening in settings universally accessed (eg, pediatric clinics, childcare settings) across diverse populations, addressing health disparities in ADHD identification and intervention.

In summary, the current study’s findings suggest that it may be possible to develop a computer-based task that could be used to screen for increased odds of developing childhood ADHD and that would have clinical utility in screening and referral to early interventions. However, there is still much work to be done, including replication of the current study findings in relation to childhood ADHD status after following these infants longitudinally to early childhood, before clinical translation is justified. Although family-based interventions during infancy to improve outcomes by reducing symptoms and impairment associated with ADHD are not currently available, there is a growing interest in this area, and research is emerging.^81^

## CRediT authorship contribution statement

**Heather M. Joseph:** Writing – original draft, Resources, Project administration, Methodology, Investigation, Funding acquisition, Data curation, Conceptualization. **Anastasia N. Kerr-German:** Writing – original draft. **Jihui L. Diaz:** Writing – original draft, Methodology, Formal analysis. **Michelle A. Wilson:** Writing – original draft, Project administration, Investigation, Data curation. **Erik Thiessen:** Writing – review & editing, Supervision, Resources, Methodology, Conceptualization. **Brooke S.G. Molina:** Writing – review & editing, Supervision, Conceptualization.
