# Supplementary material for: Infant Habituation Task Performance Is Associated With Familial Risk for Attention-Deficit/Hyperactivity Disorder
Source: JAACAP Open. 2026 Jun 17;4(5):796–808. doi: 10.1016/j.jaacop.2026.06.003 (PMC13643470; doi:10.1016/j.jaacop.2026.06.003)
Supplement: Supplementary file 1 [file mmc1.docx]

**Table S1.** Multivariate Logistic Regression: Prediction of ADHD familial risk status controlling for infant sex and parent education

|  | | OR (95% CI) | p-value |
| --- | --- | --- | --- |
| Unimodal | |  |  |
|  | Infant Sex (Female vs Male) | 0.96 (0.47, 1.97) | 0.913 |
|  | Parent Education |  |  |
| 4-year degree vs Less than a 4-year degree | | 1.22 (0.42, 3.54) | 0.712 |
| Graduate degree vs Less than a 4-year degree | | 0.87 (0.34, 2.22) | 0.768 |
|  | Unimodal Classes |  |  |
| Class 2 vs 1 | | 0.81 (0.27, 2.31) | 0.690 |
| Class 3 vs 1 | | 2.00 (0.74, 5.81) | 0.181 |
| Multimodal | |  |  |
|  | Infant Sex (Female vs Male) | 1.05 (0.52, 2.12) | 0.890 |
|  | Parent Education |  |  |
| 4-year degree vs Less than a 4-year degree | | 1.28 (0.43, 3.87) | 0.663 |
| Graduate degree vs Less than a 4-year degree | | 1.01 (0.38, 2.73) | 0.984 |
|  | Multimodal Classes |  |  |
| Class 2 vs 1 | | 2.46 (0.91, 7.46) | 0.089 |
| Class 3 vs 1 | | 4.50 (1.36, 16.51) | **0.017** |

Note: OR = Odds ratio; CI = Confidence Interval. Values in bold indicate *p* < .05.
